# The lepidopteran analyst: how caterpillars, moths and butterflies encode taste identity and valence

**DOI:** 10.1007/s00359-025-01788-9

**Published:** 2025-12-29

**Authors:** Dimitri Peftuloglu, Joop J. A. van Loon, Alexander Haverkamp

**Affiliations:** Laboratory of Entomology, Wageningen University & Research, 6708PB Wageningen, The Netherlands

**Keywords:** Insect gustation, Plant metabolites, Associative learning, Taste coding, Sensory evolution

## Abstract

The sense of taste is crucial for butterflies and moths to accomplish important life tasks such as feeding or selecting suitable oviposition sites. In Lepidoptera taste is of special importance since they are constantly confronted with a vast amount of plant secondary metabolites combined with sugars, amino acids and other primary metabolites that they need to fuel their metabolism. The high importance of many tastants for feeding and oviposition gives these compounds a strong innate meaning for the animal. During associative learning this positive or negative valence often functions as reward or punishment, giving the sense of taste an important role during memory formation. In this review we first address some general mechanisms of gustatory detection before focusing on the taste system of caterpillars and adult Lepidoptera specifically. We list recent examples of receptor genes for which the main ligands have been identified, but place special emphasis on the neuronal and behavioral responses to different tastants. Thereafter the detection of primary and secondary metabolites is reviewed, with a focus on the role of secondary plant metabolites during host-plant choice. Finally, we compiled different results on the taste processing in the lepidopteran brain and highlight the role of taste during associative learning. In this review we combined information on the role of taste for both innate and learned responses of Lepidoptera to their environment, aiming to provide a starting point for further explorations into this essential sensory modality.

## Introduction

The sense of taste plays a key role in all animals when it comes to discriminating nutritious from toxic foods. In insects, taste is also used in selecting oviposition sites and during mate recognition. All this is achieved through the use of a finely tuned gustatory apparatus, which, upon contact with a given substrate, will quickly allow the animal to evaluate its suitability (Wang and van Loon 2023; Xu 2020; Zhang et al. 2024). In humans taste perception is often categorized into five distinct modalities (sweet, sour, salty, bitter and umami), while in insects, where taste sensation has been addressed mostly on a physiological level, the diversity of chemicals with a specific valence for the behavior of the animal is higher (Liman et al. 2014; Reiter et al. 2015; Peftuloglu et al. 2024). Lepidoptera are well suited to explore this complex taste coding in insects, because their herbivorous lifestyle and the high degree of host-plant specialism make them respond to a large array of plant secondary metabolites. In particular the detection of plant compounds characteristic for the host-plant of a certain species, so-called token stimuli, has been studied in much detail for many Lepidoptera (Wang and van Loon 2023). Moreover, the high metabolic demands of the larval caterpillar stage as well as of some adult moths also make lepidopterans an interesting model to study diet selection with regards to primary metabolites (Glendinning et al. 2007; Kelber 2003). Finally, because caterpillars as well as adult butterflies and moths occupy very different ecological niches, lepidopterans make it possible to study physiological adaptations of different life stages within the same animal species to very different environments (Zhang et al. 2024). In the last century, a substantial number of behavioral, neurophysiological, morphological and molecular studies have contributed to the current understanding of the lepidopteran gustatory system. This review aims to cover three main topics: after a general introduction on the insect taste system, detailed descriptions of juvenile and adult lepidopteran taste organs are presented. Subsequently, reports on how both larval and adult Lepidoptera detect primary and secondary plant metabolites will be reviewed. Lastly, attention will be paid to how taste information is processed from the periphery to higher brain centers, and how detection of ecologically meaningful gustatory stimuli is involved in cognitive processes such as associative learning.

## Insect gustation

In insects, taste detection is performed by specific structures named sensilla, located on several body appendages, such as mouthparts, legs, ovipositor and antennae. Taste sensilla contain an inner, hollow lumen that houses a variable number of dendrites extending from the cell body of gustatory sensory neurons (GSNs) (Kvello 2024). These neurons are surrounded by several auxiliary cells, which provide the adequate ionic composition within the sensillum lumen necessary for GSNs functionality, and at the base of the sensillum one mechanosensory neuron is found (Gödde and Krefting 1989; Ma and Schoonhoven 1973). The often hair-like sensilla present a single pore at the top, allowing tastants to enter the sensillum lumen and to reach the dendrites of the GSNs.

Within the sensillum lumen GSNs are found, whose dendrites express GRs at their terminal endings. These receptors are not G-protein coupled receptors with secondary messengers, but ion channels (Gomes et al. 2024). Sensory neurons found in taste sensilla are surrounded by what is a called a “dendritic sheath”, which divides the sensillum into a sensillar chamber and a dendritic chamber (Mitchell et al. 1999). The function of this sheath, which is particularly prominent in taste sensilla, is to provide protection and structural support to the dendrites of GSNs. Gustatory receptors expressed on GSNs can be tuned to primary plant metabolites that stimulate feeding activity such as mono/oligosaccharides (i.e. glucose, fructose, sucrose and myo-inositol), amino acids and salts, but can also be tuned to secondary plant metabolites which can be used by a feeding or ovipositing insect to locate a substrate suitable for growth and development (either its own or of its offspring). At the same time, some secondary metabolites can have deterrent effects on the feeding activity of some herbivores, hindering development in growing juveniles. The GRs tuned to these deterrent compounds have a much greater sensitivity for their ligands in comparison to GRs tuned to plant primary metabolites or to the ones tuned to token stimuli (Chapman 2003), allowing an insect to avoid feeding on substrates that might severely hinder growth or might even be lethal. Often this sensitivity is more pronounced in specialist feeders than in generalists, suggesting a greater capacity of dealing with deterrents in the latter group, most likely with the aid of post-ingestive mechanisms (Chapman 2003).

Upon contact with a given substrate, gustatory cues will enter the sensillum lumen through the apical pore. The taste molecule will then diffuse through the sensillum lymph and finally interact with the binding pocket of one of the gustatory receptors expressed in the GSNs dendrite. If the taste molecule matches the overall shape and chemical properties of the receptor binding pocket it will induce a conformational change, opening a channel that will allow for an influx of ions, also called a receptor potential. This travels downstream to the cell body, triggering an action potential in the GSNs (Gomes et al. 2024; Morinaga et al. 2022; Frank et al. 2024). The action potential then travels down the GSNs axon towards the primary center for processing of taste information in the brain, namely the suboesophageal ganglion (SOG) (Jørgensen et al. 2006). In the larvae of the moths *Helicoverpa armigera* (Hübner; Lepidoptera: Noctuidae) and *Manduca sexta* (Linnaeus; Lepidoptera: Sphingidae) the axons of the GSNs within the galeal styloconic sensilla innervate the SOG through the maxillary nerve in the ipsilateral region of the maxillary neuromere (Tang et al. 2014; Kent and Hildebrand 1987). Lastly, information from the SOG is sent to higher brain regions through projection neurons, where it is processed, and according to the nature of the gustatory stimulus detected (i.e. phagostimulant vs. feeding deterrent) an appropriate behavioral response will take place.

## Taste organs of larval lepidoptera

**Fig. 1:**
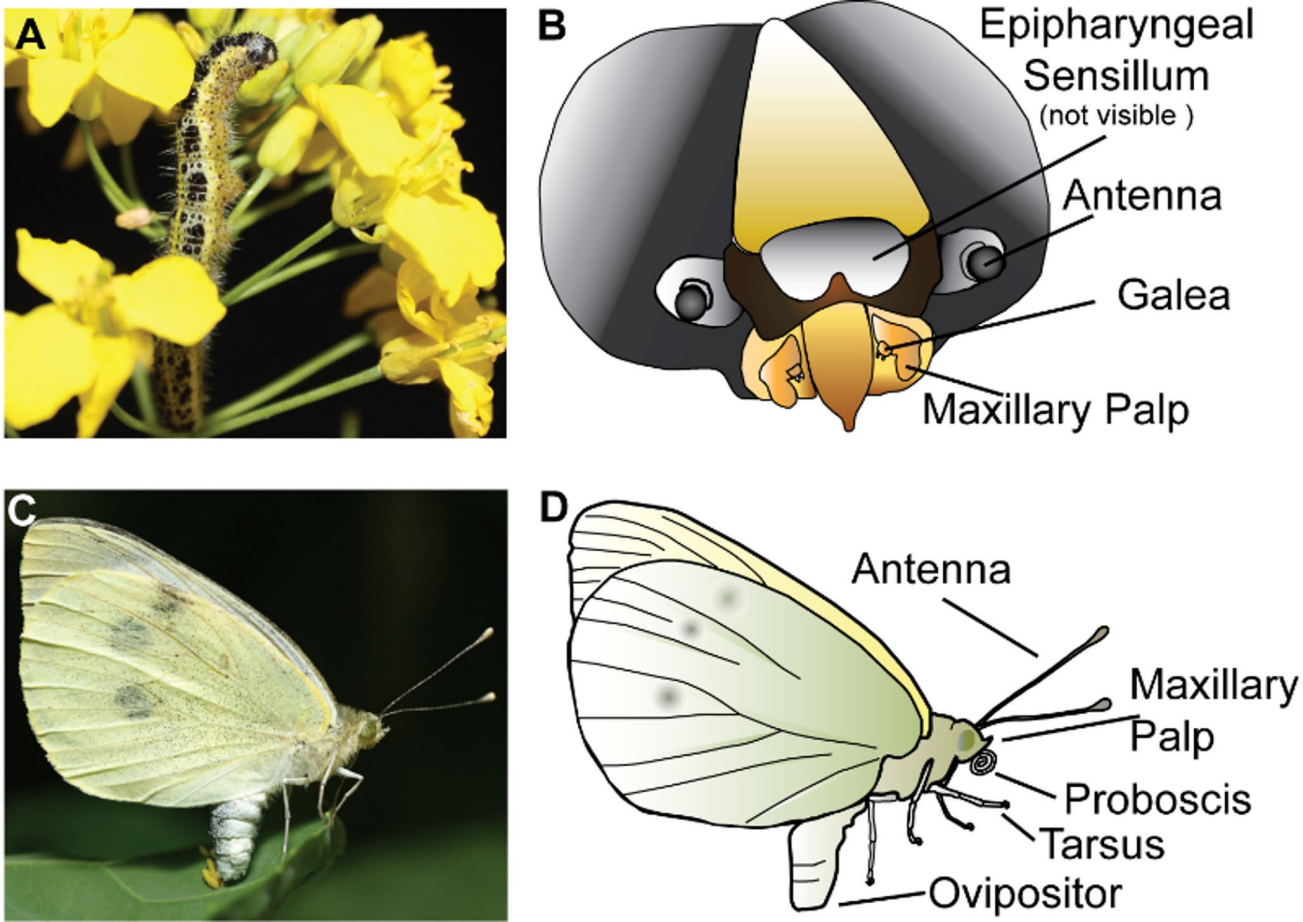
Major taste organs in caterpillars and adult Lepidoptera. **A** *Pieris brassicae* caterpillar feeding on the flowers of *Brassica napus.* **B** Schematic drawing of a caterpillar head (frontal view) showing the main taste organs. **C** Female *P. brassicae* butterfly ovipositing on a *Brassica oleracea* leaf. **D** Schematic drawing of a butterfly showing the main taste organs. Note that other body parts of the butterfly might also carry gustatory sensilla, however, their function has not yet been explored

Caterpillars present a relatively simple yet very effective gustatory apparatus, that allows them to successfully accomplish one of the main tasks of this life stage, namely feeding. Food intake, which occurs at very high rates, is achieved through the use of biting-chewing mouthparts, which include mandibles, maxillae, labrum and labium (i.e. upper and lower lips). In caterpillars, the appendages bearing gustatory sensilla are the maxillae, the epipharynx, and for some species, even the antennae (Schoonhoven and van Loon 2002) (Fig. 1A, B).

Maxillae are structures located externally below the mandibles at both sides of the oral cavity, consisting of a basal part from which two distinct lobes depart. The inner (or medial) lobe is called a galea, and the outer (or lateral) lobe is called a maxillary palpus. Caterpillar galeae normally present two taste sensilla, whose GSNs can express GRs tuned to both primary and secondary plant metabolites, among which feeding deterrents. For instance, larvae of the gypsy moth *Lymantria dispar* (Linnaeus; Lepidoptera: Erebidae) bear taste sensilla on their galeae expressing GRs sensitive to inositol as well as GRs sensitive to feeding deterrents (such as caffeine and strychnine), and to inorganic salts like potassium chloride (Martin and Shields 2012). In addition to this, experiments on *Estigmene acrea* (Drury; Lepidoptera: Arctiidae) highlighted that lateral galeal sensilla present one GR with very high sensitivity for pyrrolizidine alkaloids, down to values of 1 picomolar. These compounds are a family of secondary metabolites used by this species for host-plant recognition (token stimulus), but also for its own defense against natural enemies, by storing it in body tissues, and lastly as precursors for sex pheromone synthesis during the adult life stage (Bernays et al. 2002). Therefore, taste detection by galeal sensilla seems to contribute in many different ways to caterpillar survival, by triggering a range of essential behaviors needed for growth and development, and experiments with caterpillars whose galeae were removed showed pronounced losses in discriminative and selective capacity (Schoonhoven and Blom 1988).

Whereas sensilla on caterpillar galeae have so far been always associated with contact chemoreception (i.e. taste detection), the picture gets more nuanced when we shift our focus to the maxillary palps. Each palpus normally bears a higher number of sensilla if compared to galeae, and of these, some display the typical single pore tip opening of taste sensilla, while some others present multiple pores, thus having an olfactory function (Shields 2021). For instance, the maxillary palps of the moth *Yponomeuta cagnagellus* (Hübner; Lepidoptera: Yponomeutidae) display olfactory sensilla housing receptor cells sensitive to plant volatiles such as benzaldehyde and hexanal (Roessingh et al. 2007). Besides these olfactory sensilla, the maxillary palp contains five taste sensilla that are innervated by about 20 neurons (Asaoka 2003). In *Bombyx mori* (Linnaeus; Lepidoptera: Bombycidae) these neurons have been found to play a crucial role during host-plant acceptance by detecting secondary plant metabolites (Tsuneto et al. 2020).

The epipharynx is the inner side of the labrum, or upper lip, and it is involved in food intake and in delivering the substrate to the oral cavity. On its inner side taste sensilla are often found, suggesting further involvement in food acceptance or rejection. In addition to the taste sensilla found on maxillary palpi and epipharynx, additional sensilla are found in some cases in the hypopharynx and possibly deeper in the oral cavity (Schoonhoven and van Loon 2002). Lastly, one taste sensillum expressing at least one GR tuned to sugars has been located on the antennae of the maize stalk borer, *Busseola fusca* (Fuller; Lepidoptera: Noctuidae), a feature that might be present in other noctuid larvae as well (Juma et al. 2008).

## Taste organs of adult Lepidoptera

Similar as in larvae, adult Lepidoptera (i.e. butterflies and moths) use taste detection to evaluate food sources, but, unlike caterpillars, the same sense is used to accomplish other crucial life tasks, such as selection of oviposition sites (Tsuchihara et al. 2022; Wang and van Loon 2023). The anatomical regions for which the presence of taste sensilla has been documented are the proboscis, the maxillary palps, the tarsi, the ovipositor and the antennae (Xu 2020) (Fig. 1C, D).

The proboscis of Lepidoptera is an elongated organ, kept coiled when not feeding, formed by the two galeae interlocked together, and it is used by adults to feed on sugar-rich fluids, such as floral nectars or fluids released by fermenting fruits. Taste sensilla housing GRs binding to sugars and alcohols have been reported for a few species (Ômura et al. 2008; Zhang et al. 2024), and such sensilla are often distributed on the distal segment of the proboscis (Faucheux 2013). This feature allows a foraging individual to rapidly evaluate the nutritional quality of the food source and to decide whether to continue feeding. At the same time, the presence of taste sensilla on the inner walls of the proboscis has been reported in a few species. For instance, *Papilio xuthus* (Linnaeus; Lepidoptera: Papilionidae) presents taste sensilla on the inner walls of each galea, and these are responsible for the detection of sodium salts during puddling behavior, i.e. the uptake of saline ions found in water puddles, used for metabolism and excretion enhancement (Inoue et al. 2012, 2015).

In contrast to the maxillary palps of the caterpillar, the palps of adult Lepidoptera have so far not been shown to contain any contact taste sensilla. However, they do house chemosensory neurons responsive to CO_2_ which project to the olfactory processing center, the antennal lobe in the brain of adult Lepidoptera (Guerenstein et al. 2004).

Several lepidopteran families bear taste sensilla on the tarsi of their legs, and the involvement of these in detection of primary and secondary metabolites has been shown for various species. In *Heliconius erato* (Linnaeus; Lepidoptera: Nymphalidae) both males and females present taste sensilla on their tarsi, but while males possess these sensilla only on the tarsi of the mid and hind legs, females also bear them on the forelegs. The former sensilla, displayed by both sexes, are involved with sugar detection and therefore with feeding, while the latter are tuned to host-plant gustatory cues and are used by ovipositing females to assess the quality of potential oviposition sites (Silva et al. 2018; Renwick and Chew 1994). Similar morphological and functional differences between sexes have been reported for other lepidopteran families (Ma and Schoonhoven 1973; Marion-Poll et al. 1992). In addition to sugars and token stimuli, some studies have reported amino acid detection in tarsal taste sensilla. For instance, one study with *H. armigera* showed that adult females bear taste sensilla on their foreleg tarsi, which house GRs tuned to sugars but also to seven amino acids (Zhang et al. 2010). While neurophysiological recordings highlighted strongest neuronal firing for both sucrose and the essential amino acid lysine, tarsal stimulation with sugars and lysine induced significantly different behavioral responses both in terms of proboscis extension reflex (PER) and in terms of length of subsequent feeding bout. In this study, most sugars induced a significantly higher number of PER events when compared to lysine, and the length in seconds of feeding bouts was almost always longer for sugars (Zhang et al. 2010). Amino acids, the building blocks of proteins, are limiting nutrients during larval growth and development, while in adult Lepidoptera these are used for nucleic acid formation, oogenesis, and to repair damaged tissues (Cahenzli and Erhardt 2013) and less frequently as metabolic fuel (Levin et al. 2017). Sugars, on the other hand, are used in daily metabolic processes and activities such as flying, exploring habitats, finding mates and ovipositing, and thus their detection could be more strongly linked with feeding activity and therefore PER events, while amino acid detection might more linked to oviposition behavior in adult lepidopterans.

The ovipositor is the organ used by insect females to lay eggs and in Lepidoptera it is formed by two valves located at both sides of the gonopore, attached to the last abdominal segment. Around it several hairs can be found, most of which are mechanoreceptors used by females to orientate the ovipositor during egg-laying (Marion-Poll et al. 1992). Through these mechanoreceptors a female can assess the physical properties of potential oviposition substrates, allowing her to reject those which do not present suitable features. In addition to this, for species laying large egg clusters, mechanoreceptors can contribute to appropriate egg positioning, avoiding the deposition of eggs in double layers. In addition, a small number of taste sensilla are also present on the ovipositor, as it is the case for *Spodoptera littoralis* (Boisduval; Lepidoptera: Noctuidae). For this species, contact chemoreceptors on the ovipositor present four GSNs expressing GRs tuned to phagostimulants and to feeding deterrents (Seada et al. 2016). It is likely that the sensory information detected by these taste sensilla contributes to oviposition site selection in this species. Similar results have also been obtained for other lepidopterans (Klijnstra and Roessingh 1986; Maher et al. 2006; Qiu et al. 1998). However, the detection of token stimuli through ovipositor taste sensilla has not been investigated extensively, and it remains uncertain to what extent these contribute to host-plant specificity. Lastly, besides gustatory and mechanosensory detection, ovipositors presenting olfactory sensilla have been described for several species (Klinner et al. 2016).

Insect antennae are the appendages bearing the greatest number of olfactory sensilla, and Lepidoptera make no exception to this. However, antennae of many lepidopteran species present also a number of taste sensilla, whose function is not always clear. In a study on three tortricid moth species the gustatory responses of antennal and labial pit taste sensilla was investigated, performing neurophysiological recordings on both sensilla types. Both sensilla types responded to the sugars fructose and sucrose, and to the salts KCl and NaCl, although the overall neuronal firing patterns were much more pronounced in the taste sensilla located on the labial pits (Amat et al. 2022). At the same time, another study on *S. littoralis* has shown that for this species antennal taste sensilla contain GSNs expressing GRs tuned to sugars, salts and water (Popescu et al. 2013), while taste sensilla on the antennal tip of the monarch butterfly, *Danaus plexippus* (Linnaeus; Lepidoptera: Nymphalidae), also responded to host-plant extracts (Baur et al. 1998).

## Plant primary metabolites and salts

Plant primary metabolites, such as carbohydrates and amino acids, are molecules synthesized by plants to sustain growth, and at the same time they are fundamental for the development of the caterpillar, as well as for body maintenance of adult Lepidoptera. While feeding on plant material an insect herbivore ingests a large quantity of carbohydrates, but also amino acids, fatty acids and moderate amounts of inorganic salts. Carbohydrates are used to sustain the energetic requirements of the body, and to perform all daily tasks such as exploratory activities, mating and oviposition. Sugars can be stored in an insect-specific organ, the fat body, where they are polymerized into glycogen, or alternatively converted to triglycerides, with obvious advantages in terms of energy storing capacity (Skowronek et al. 2021). The fat body also synthesizes most of the hemolymph proteins, for which the amino acids that are acquired through the diet serve as building blocks (Skowronek et al. 2021).

On a molecular level, Lepidoptera detect carbohydrates with two main classes of sugar receptors: those with sequences similar to the *Drosophila* receptor DmelGr43a which are mainly tuned towards fructose, and those closer to the *Drosophila* receptor DmelGr64a which bind sucrose (Ma et al. 2024) (Table 1). Interestingly, some of these receptors not only respond to sugars and sugar alcohols such as inositol, but also to plant secondary metabolites such as synephrine or isoquercitoline (Endo et al. 2024; Ozaki et al. 2011). In contrast to sugars little is known about the molecular mechanisms of amino acid detection in Lepidoptera and only one receptor has so far been identified responding to proline (Xu et al. 2016). These molecular mechanisms have received much attention recently and have been reviewed in detail (Sato 2024; Xu 2020). Below we will therefore mostly focus on the physiological and behavioral response to different primary metabolites in caterpillars and adult Lepidoptera.

**Table 1:** Gustatory receptor genes for which the main ligands have been identified in different Lepidoptera

Ligand class	Ligands	Function	Species	Receptor gene	References
Sugar	Fructose (isoquercetin, chlorogenic acid)	Feeding stimulant	*Bombyx mori*	BmorGr9	(Endo et al. 2024; Gomes et al. 2024)
Sugar	Fructose	Feeding stimulant	*Helicoverpa armigera*	HarmGr4	(Jiang et al. 2015)
Sugar	Fructose	Feeding stimulant	*Plutella xylostella*	PxylGR43a-1 PxylGR43a-2	(Liu et al. 2020)
Sugar	Fructose	Feeding stimulant	*Spodoptera litura*	SlitGr8	(Liu et al. 2019)
Sugar	Myo-Inositol	Feeding stimulant	*Bombyx mori*	BmorGr8	(Zhang et al. 2011)
Sugar	Myo-Inositol, epi-inositol	Feeding stimulant	*Bombyx mori*	BmorGr10	(Kikuta et al. 2016)
Sugar	Sucrose, fructose, fucose	Feeding stimulant	*Helicoverpa armigera*	HarmGr6	(Zhang et al. 2024)
Sugar	Sucrose	Feeding stimulant	*Helicoverpa armigera*	HarmGr10	(Zhang et al. 2024)
Sugar	Sucrose, fructose, sorbitol	Feeding stimulant	*Ostrinia furnacalis*	OfurGr43a	(Shi et al. 2024)
Amino acid	Proline	Feeding stimulant	*Helicoverpa armigera*	HarmGr195	(Xu et al. 2016)
Sec. metabolite	Caffeine, coumarin	Feeding deterrent	*Bombyx mori*	BmorGr16	(Kasubuchi et al. 2018)
Sec. metabolite	Coumarin, caffeine, pilocarpine	Feeding deterrent	*Bombyx mori*	BmorGr19	(Kasubuchi et al. 2018)
Sec. metabolite	Coumarin, caffeine	Feeding deterrent	*Bombyx mori*	BmorGr53	(Kasubuchi et al. 2018)
Sec. metabolite	Coumarin, (sinigrin, strychnine)	Feeding deterrent	*Helicoverpa armigera*	Harm180	(Chen et al. 2022)
Sec. metabolite	Brassinolide, 24-epibrassinolide	Feeding deterrent	*Plutella xylostella*	PxylGr34	(Yang et al. 2020)
Sec. metabolite	Isoquercitoline, chlorogenic acid	Host-plant stimulus	*Bombyx mori*	BmorGr6	(Endo et al. 2024)
Sec. metabolite	Isoquercitoline	Host-plant stimulus	*Bombyx mori*	BmorGr63	(Zhang et al. 2022)
Sec. metabolite	Unknown	Host-plant stimulus	*Bombyx mori*	BmorGr66	(Zhang et al. 2019)
Sec. metabolite	Sinigrin	Host-plant stimulus	*Pieris rapae*	PrapGr28	(Yang et al. 2021)
Sec. metabolite	Synephrine	Host-plant stimulus	*Papilio xuthus*	PxutGr1	(Ozaki et al. 2011)

### Carbohydrates

Larval lepidopterans are likely to encounter carbohydrates directly on the substrates on which mothers have laid the eggs from which they hatch. Sugars such as glucose, fructose and sucrose are produced by plants as products of photosynthesis and these are stored in plant tissues like roots, stems and leaves, and very often caterpillar sensilla show high neuronal responses to the most common sugars found in plant tissues. For instance, sucrose is detected by the galeal sensilla styloconica of the caterpillars of the butterfly *Atrophaneura alcinous* (Klug; Lepidoptera: Papilionidae), and, interestingly, a very similar response is observed in the sensillum located on the epipharynx, pointing to the involvement of this sensillum in food acceptance in this species (Tsuchihara et al. 2022). The galeal sensilla of *Ostrinia furnacalis* caterpillars (Guenee; Lepidoptera: Crambidae) show dose-dependent responses to sucrose and fructose, sugars that are commonly found in several Poaceae on which these larvae feed. Moreover, in a study on this species it was observed that the sensitivity of peripheral sensilla to these compounds, and subsequently the feeding preferences displayed, were strongly correlated to the expression of Neuropeptide F in the insect haemocoel (Wang et al. 2021), a signalling molecule found in invertebrates which is involved in the modulation of food seeking and feeding behavior (Fadda et al. 2019). By injecting caterpillars with a double-stranded Neuropeptide F coding fragment the expression of the putative gene was knocked-down, and in subsequent tests treated animals displayed significantly lower neurophysiological and behavioral responses when compared to control, non-injected caterpillars. Given that the gene encoding for Neuropeptide F is highly conserved among insects and other invertebrates (Fadda et al. 2019), it is likely that similar regulatory mechanisms occur in other lepidopteran species.

Besides sucrose, fructose and glucose, other carbohydrates induce strong neuronal responses in Lepidoptera, such as inositol. This sugar-alcohol has several functions within plant tissues, including cell wall biosynthesis, cell membrane integrity and as secondary messenger in several biosynthetic pathways (Loewus and Murthy 2000). In *B. mori* the receptor BmorGr10 detecting two stereoisomers of this molecule, namely myo- and epi-inositol, is expressed in the sensilla of galea, maxillary palps and even epipharynx (Table 1). For this species, myo-inositol detection induces sustained feeding in foraging caterpillars, enhancing growth rates and shortening egg-to-pupa developmental time (Kikuta et al. 2016). In the same study, neurophysiological responses of the mentioned receptor were investigated through heterologous oocyte expression and subsequent stimulation with increasing concentrations of myo-inositol. Results showed that the minimum concentration needed to evoke current was 1.9 mM, a value much lower than the one observed for mulberry leaf extracts (*B. mori*’s main host-plant), hinting to the ecological relevance of this compound for growing *B. mori* caterpillars.

Adult Lepidoptera feed on sugar-rich substrates, such as floral nectar or fluids released by rotting fruits (Bruinsma et al. 2014; Haverkamp et al. 2016; Ômura et al. 2008), and there is a large body of literature presenting behavioral responses to sugars such as sucrose, glucose and fructose, as well as a moderate number of neurophysiological studies presenting neuronal responses to these compounds (Romeis and Wäckers 2000; Zhang et al. 2010, 2024; Su et al. 2020). Sugars are detected at the periphery with sensilla located on the proboscis, and in some cases labial palps, tarsi and antennae (Amat et al. 2022; Guo et al. 2018). One interesting aspect to observe is that for at least some species sugar detection during larval and adult stages occurs with different gustatory receptors. This is the case, for instance, in the moth *H. armigera*, whose caterpillars detect sucrose mainly with the HarmGr10 receptor expressed in the galeal styloconic sensilla, while adults of the same species primarily use the HarmGr6 receptor, expressed in the proboscis, tarsi and antennae (Table 1). Both receptors can detect several saccharides, but HarmGr10 shows a much higher affinity to sucrose when compared to HarmGr6 (Zhang et al. 2024). Neurophysiological recordings highlighted that for the caterpillar receptor HarmGr10 sucrose concentrations as low as 0.1 mM suffice in eliciting neuronal responses, while for the moth receptor HarmGr6 concentrations of at least 10 mM are needed. One possible explanation for these discrepancies lies in the different chemical profiles of the food sources that the two life stages use. While sucrose concentrations in nectars are often high, plant tissues on which caterpillars feed contain much lower sucrose concentrations that the caterpillar peripheral sensory system should be capable to detect, allowing them to exploit all available food sources.

### Amino acids

Amino acids are a class of organic compounds vital for protein synthesis, and they are therefore essential for growth, body maintenance and reproduction. GRs involved in amino acid detection can sometimes present a non-specific ligand capacity, meaning that they can detect both amino acids and other primary metabolites, such as sugars. One of the first proofs for amino acid detection by larval Lepidoptera was reported in 1969 for *Pieris brassicae* (Linnaeus, Lepidoptera: Pieridae) caterpillars, exhibiting strong neurophysiological responses to amino acids from one GSN located on a maxillary styloconic sensillum. In addition to that, stimulation with solutions containing both amino acids and sugars displayed neuronal activity from two distinct cells, indicating the presence of an amino acid sensing receptor within the larval maxillae (Schoonhoven 1969).

In adult Lepidoptera, amino acids are relevant primarily for reproduction. Amino acids are found in floral nectars (Nicolson 2022) and butterflies are likely to ingest them during foraging. For both sexes, several studies have shown a positive correlation between amino acid uptake and reproductive traits such as male spermatophore quality and female fecundity. In a study on the butterfly *Coenonympha pamphilus* (Linnaeus; Lepidoptera: Nymphalidae) adult males were fed on nectar mimics either enriched with amino acids or not, and were subsequently allowed to mate. In a later moment, females were allowed to oviposit, and eggs laid by individuals that mated with males fed on amino acid-enriched nectars gave birth to caterpillars of significantly higher hatching mass, as compared to individuals who mated with males fed on nectars without amino acids. This phenomenon hints to the possibility that amino acids obtained during adulthood are transferred during mating as nuptial gifts, and that females can ultimately convey these nutrients directly to the progeny (Cahenzli and Erhardt 2013). In another study with the butterfly *Araschnia levana* (Linnaeus; Lepidoptera: Nymphalidae) larvae were either fed on natural or nitrogen-enriched diets and allowed to pupate. Females emerged were subsequently fed with nectar mimics, either enriched with amino acids or not. Females originating from caterpillars fed on natural diets laid significantly more eggs when their adult food included amino acids, while for those originating from caterpillars fed on nitrogen enriched diets these differences were not found. These results suggest that, in natural circumstances, female butterflies can compensate amino acid deficiencies due to sub-optimal larval feeding through nectar foraging, and most importantly, that this uptake can lead to higher fecundity in the mated individual (Mevi-Schütz and Erhardt 2003). Additionally, at least some adult Lepidoptera can use nectar amino acids as metabolic fuel during rest. In a study on the hawkmoth *M. sexta* animals were fed nectar mimics containing isotopically labeled amino acids and subsequently placed in sealed chambers where a constant airflow was pumped in and out. Analysis of the outflow of air containing also the products of respiratory activity highlighted presence of the ^13^C label, indicating that these compounds can, in some circumstances, be used as energy source (Levin et al. 2017).

Lastly, while discussing the relevance of amino acids in adult Lepidoptera, one genus worth mentioning is *Heliconius* spp. Like most adult lepidopterans, members of the genus feed on floral nectar, but at the same time, they display the unique behavior of actively collecting pollen from flowers which is subsequently mechanically crushed and enzymatically degraded to amino acids. These are often used as nuptial gifts by males during mating (Cardoso et al. 2009), and of these, the essential fraction can be transferred to eggs by mated females (O’Brien et al. 2003). At a molecular level, this genus presents a great diversification of gustatory receptors in all taste organs, and some of these receptors are found uniquely within members of the genus, suggesting a possible involvement in the unique pollen feeding behavior (Briscoe et al. 2013).

### Salts

Inorganic salts, or more appropriately, the cations originating from the ionic dissociation of these compounds, are of paramount importance for a range of biological processes taking place within insect cells and tissues. For instance, sodium ions are found in the fluids immediately surrounding neurons and actively contribute to signal transduction across nerves (Dethier 1977), and, considering the importance of such ions, a great number of insects possess salt receptors in their taste sensilla (Agnihotri et al. 2016; Inoue et al. 2012). Larval lepidopterans are likely to acquire salts primarily through their plant diet, and the only way to compensate for the very low concentrations of these compounds within plant tissues is through sustained and prolonged feeding activity. As for adult lepidopterans, several species ingest salts through the already mentioned puddling behavior, where individuals can be observed to drink from mud puddles. Such waters contain salt ions which are subsequently stored in the body, and can be used to sustain different biological processes, such as flight activity. For instance, in a study on *H. armigera* individuals fed with a sodium-deficient diet displayed significantly slower flight activity when compared to individuals fed on a sodium-rich one (Xiao et al. 2010). Considering that butterflies need to fly to locate mates and reproduce, salt intake might affect fitness.

## Plant secondary metabolites

Nearly all lepidopteran species are herbivores feeding on plant material at some stage in their life (Wiens et al. 2015) and most species show a degree of “feeding specialization”, i.e. the capacity of using only a few chemically (and therefore also taxonomically) related plant families as feeding substrates. The reasons for the dominance of specialized feeding lifestyles have been discussed in the past, and to this day, the most accredited hypotheses include less time needed to locate and select food sources (i.e. less time exposed to risks such as predation) and less energy needed to evaluate multiple substrates as the insect can focus on specific plant metabolites for detecting their host-plant ultimately translating into higher shares of energy to be allocated to other key aspects of an insect’s life, such as mating and oviposition (Bernays 1988, 2001).

All specialist herbivores present the capacity to identify their own host-plants through specific molecules found in the plant’s tissues, called token stimuli. Such molecules are secondary metabolites usually synthesized by the plant for its own defense, but through evolutionary times specialized insects have evolved to exploit these compounds for their own benefit. This detection of token stimuli by a specialized herbivore allows for the identification of an optimal feeding or oviposition site, ultimately granting species survival. On a molecular level several token stimuli receptors have been identified, such as PrapGr28 detecting the glucosinolate sinigrin in *P. rapae*, PxutGr1 detecting the alkaloid synephrine in *P. xuthus* or BmGr6 responding to isoquercitoline and chlorogenic acid in *B. mori* (Table 1). Notably many of these receptors appear not to be as specific as generally assumed, indicating that the strong behavioral responses to these compounds is rather a response generated in the nervous system based on several different inputs than the result of a single receptor and a labeled-line neuronal pathway (Tsuneto et al. 2020).

The term plant secondary metabolite includes the wide range of compounds that do not directly sustain growth, but that are actively involved in plant–environment interactions. Molecules belonging to this category thus confer specific chemical characteristics to plant tissues, allowing a given species to increase its chances of reaching the reproductive stage and seed set. Some plant secondary metabolites for example confer particular scents to attract insect pollinators and thus increase genetic variability between individuals of a plant population.

A great number of secondary metabolites, however, exert a repellent effect on phytophagous animals by giving a bitter or noxious taste to plant tissues (Jeschke et al. 2021), while others limit feeding by sticking to chewing mouthparts, hindering normal biting activity (Betz et al. 2024). In both cases, this can limit herbivore damage and increase chances of reaching plant reproductive stage. Considering that insects represent the biggest group of herbivores, and that Lepidoptera almost exclusively feed on plant material during the larval stages, studying how plant secondary metabolites are detected, and which neurophysiological and behavioral responses these exert can greatly contribute to increase our understanding of taste processing in this ecologically relevant insect order. In addition to this, through evolutionary times oligophagous and monophagous insects have gained the capacity to tolerate and even exploit specific secondary metabolites belonging to the plant families on which they feed to their own benefit. In such cases, a compound which exerts a repellent effect on a given insect species could have the opposite effect on another one, stimulating feeding activity and oviposition (Wang and van Loon 2023). These compounds allow researchers to study co-evolutionary interactions between plants and herbivores and to infer when particular herbivore–host-plant relationships have first occurred in evolutionary time.

### Glucosinolates

One family of plant secondary metabolites which has been studied extensively, especially in relation to insect–plant interactions, is the one of glucosinolates (GSL). These are sulfur- and nitrogen-containing compounds synthesized from amino acids which are converted into toxic products upon herbivory, and are produced, among others, by plants belonging to the Brassicaceae, Capparaceae and Resedaceae families (Mithen et al. 2010). These compounds are non-toxic until brought together with the enzyme myrosinase, which is stored in a separate cell compartment. During herbivore feeding the myrosinase then hydrolyses the non-toxic GSL into several toxic products, among which isothiocyanates and nitriles, and for many generalist herbivores these exert a strong deterrent effect, limiting feeding (Hopkins et al. 2009).

However, it is important to stress that even for generalist herbivores GSL products often do not exert lethal effects, and their action mostly limits feeding activity, with obvious consequences for growth rates and larval mass. For instance, a study with the generalist *H. armigera* showed that individuals allowed to feed on cabbage and kale (both brassicaceaous plants containing GSL) reached pupation regardless of the GSL in the diet, but larval and pupal weight were negatively affected by GSL content and developmental time was prolonged. For some generalist lepidopterans, including *H. armigera*, a part of the GSL ingested can be detoxified through conjugation with gluthatione and excretion of conjugates in the faeces (Jeschke et al. 2021). Specialist lepidopterans, however, have developed more effective strategies to overcome the toxic effects of GSL products. This is the case for the species of the Pieridae family that rely on GSL to locate feeding and oviposition sites (Wang and van Loon 2023). A specific protein, NSP (nitrile specifier protein) is released into the gut lumen and interferes with the action of myrosinases, forming non-toxic conjugates with GSL that are later excreted (Okamura et al. 2019). In addition to NSP genes, also major allergen (MA) proteins play a role in overcoming GSL in plants by pierid caterpillars. These proteins, have a structure similar to NSP genes and are related to the single domain MA-proteins found in the gut of all Lepidoptera. A recent study on *Pieris rapae* (Linnaeus; Lepidoptera: Pieridae) revealed that NSP and MA gene expression varies in larvae according to the GSL profile ingested through the diet, i.e. host-plant species. This suggests that the differential expression of several adaptive genes enables *P. rapae* to feed on chemically diverse GSL-containing plants (Okamura et al. 2019). Interestingly, NSP genes have been found only in members of the Pieridae family, and likely played a crucial role in the radiation of the family. Other brassicaceous feeding Lepidoptera have developed alternative ways of dealing with GSL. For instance, the specialist moth *Plutella xylostella* (Linnaeus; Lepidoptera: Plutellidae) lacks NSP genes but still succeeds in feeding on GSL-containing plants thanks to a sulfatase which converts ingested GSL to desulfoglucosinolates. Since these compounds are not substrates for myrosinases no toxic GSL derivatives are formed, allowing larvae to grow and develop on GSL-containing plants (Chen et al. 2023). These different strategies of dealing with GLS have likely also led to specific preferences for certain GLS profiles among herbivores feeding on GLS-containing plants.

The phagostimulant activity exerted by GSL in brassicaceous feeding lepidopteran larvae was first discovered in 1910 (Verschaffelt 1910), and since then several other studies have demonstrated this (Wang and van Loon 2023; Du et al. 1995; Chew and Renwick 1995). For many Pieridae, peripheral detection of these token stimuli occurs through GSNs expressing GSL receptors housed in larval galeal sensilla styloconica (Wang and van Loon 2023), inducing feeding activity. Interestingly, not only Brassicaceae specialists present GSNs responding to GSL. In a study with two generalist lepidopterans, *Trichoplusia ni* (Hübner; Lepidoptera: Noctuidae) and *Mamestra configurata* (Walker; Lepidoptera: Noctuidae) sensillar stimulation with sinigrin elicited strong responses in galeal GSNs (Shields and Mitchell 1995), and subsequent behavioral observations highlighted how sinigrin would act as deterrent only at specific concentrations, as well as a significantly less pronounced deterrence when sinigrin was mixed with sucrose and inositol, suggesting that sugar detection might contribute to override deterrence by sinigrin (Shields and Mitchell 1995). However, it is important to note that, while brassicaceous specialist Lepidoptera present receptors responding only to GSL, responses in generalists occur through deterrent receptor cells detecting several other secondary plant metabolites (Chen et al. 2022). Knowledge on how information related to token stimuli is transmitted to higher nervous centers and the brain is scarce. In a recent study with *H. armigera* it was observed that information pertaining to phagostimulant cues (i.e. sucrose) and deterrent cues (i.e. sinigrin) was often innervating the same regions in the SOG and stimulated the same second-order neurons, suggesting that across-fiber patterns of information transmission are more likely to occur than labeled line patterns for this species, even for types of stimuli with such different meanings (Sun et al. 2022).

As for adult lepidopterans, GSL detection occurs primarily through taste sensilla located in the tarsi (Städler et al. 1995; Ma and Schoonhoven 1973; Badenes-Pérez 2023), and their detection is primarily related with oviposition site selection. Moreover, species-specific differences in GSL preference have been observed between species of Brassicaceae-feeding Lepidoptera. For instance, in a study on both *P. xylostella* and *P. rapae*, oviposition instances occurred more often on plants with high indolic GSL content, as opposed to plants rich in aliphatic GSL, although no significant correlation between oviposition preference and performance was observed (Badenes-Pérez 2023).

### Cardenolides

Another family of plant secondary metabolites that have been studied extensively is the one of cardenolides. These compounds are synthesized in several plants families, such as Asclepiadaceae and Brassicaceae (Agrawal et al. 2012; Alani et al. 2021), and the main effect exerted is the disruption of Na^+^/K^+^-ATPase, an enzyme and ion carrier responsible for maintaining membrane potentials and thus favoring signal transmission across the nervous system (Agrawal et al. 2012). Upon detection, these molecules act as feeding and oviposition deterrents for a wide range of herbivores, including several lepidopterans. In a study on both *P. rapae* and *Pieris napi oleracea* (Linnaeus: Lepidoptera: Pieridae) mated females were deterred from ovipositing on cabbage plants treated with 11 different cardenolides, although the same study showed species-specific differences in oviposition deterrence, with *P. napi oleracea* displaying higher tolerance towards these plant secondary metabolites (Huang and Renwick 1994). In addition to this, a neurophysiological and behavioral study on *Pieris* caterpillars allowed to identify two distinct deterrent chemoreceptors located in the maxillary styloconic sensilla. On a physiological level, these displayed very different sensitivities to the cardenolides tested, one showing a very high detection threshold, and the second leading to neuronal firing in response to much lower cardenolide concentrations (van Loon and Schoonhoven 1999). Considering that some brassicaceous species synthesize cardenolides during their life cycle (Alani et al. 2021), it is very likely that specialist lepidopterans feeding on this plant family have evolved receptors that allow individuals to detect very minute amounts of cardenolides co-occurring with GSL, ultimately increasing chances of reaching the adult life stage.

However, some Lepidoptera such as the monarch butterfly, *D. plexippus*, are able to avoid the toxic effects of cardenolides and even use plants containing cardenolides as their main host-plants. The main mechanism that allows *D. plexippus* larvae to feed on such toxic plants lies in the lower susceptibility of their Na^+^/K^+^-ATPase enzyme to cardenolides, as opposed to the Na^+^/K^+^-ATPase found in non-adapted herbivores. Sequence comparisons between the two enzymes, allowed to identify a minute difference (three amino acids) at the level of the alfa subunit, rendering monarch caterpillars much less susceptible to cardenolides when compared to other herbivores (Karageorgi et al. 2019). Interestingly, there is currently no direct evidence for an oviposition stimulating effects of cardenolides in this species (Baur et al. 1998), even though monarch females only lay eggs on cardenolide-containing plants and tend to prefer plants with an intermediate cardenolide content (Agrawal et al. 2021). This suggests that butterflies can detect these compounds (or correlated plant metabolites) with some of their taste receptors. In addition to this, monarch caterpillars are not only able to feed on cardenolide-containing plants, but they also actively sequester such compounds for defense against natural enemies. For instance, it was demonstrated that *D. plexippus* pupae originating from caterpillars fed on cardenolide-rich plants are more likely to be rejected by ovipositing parasitic wasps than pupae originating from caterpillars fed on cardenolide-poor plants (Stenoien et al. 2019). Moreover, cardenolide sequestration appears to be a highly plastic phenomenon to which foraging caterpillars can adapt according to internal need and foraging substrate. Caterpillars who fed on cardenolide-poor plants during their first instars can compensate by sequestering greater amounts in the last larval stages before pupation and actively seek out plant saps containing high concentrations of cardenolides (Betz et al. 2024).

### Pyrrolizidine alkaloids

An additional class of secondary metabolites that has been studied extensively in the context of plant–Lepidoptera interactions are the pyrrolizidine alkaloids (PA), compounds synthesized in plants belonging to the families of the Boraginaceae, Solanaceae and Asteraceae among others. Feeding deterrence by some PA in Lepidoptera was demonstrated on juveniles of the moth *Choristoneura fumiferana* (Clemens; Lepidoptera: Tortricidae), where individuals strongly refused to feed when the PA, senkirkine, was added to their diet (Bentley et al. 1984). However, some lepidopteran families have evolved the capacity of detoxifying these compounds, as well as storing them in their body for defense purposes. A good example is given by the moth *Estigmene acrea*, whose larva presents a receptor tuned to PA in their maxillary taste sensilla (Bernays et al. 2002). The same larvae display the capacity of detoxifying these compounds through enzymatic oxidation, and the products of these reactions are subsequently used to form novel insect-derived alkaloids, which confer unpalatability to predators. Lastly, males of this species use PA derivatives as precursors during courtship pheromone synthesis. Interestingly, larvae used in the study were almost never capable of reaching the pupal stage if fed exclusively on PA-containing plants, suggesting that for this species sensitivity to PA is not primarily correlated with feeding activity, but rather with predation avoidance and mating success (Hartmann et al. 2005). PA can also be used as token stimuli by mated lepidopteran females, like in the case of the specialist butterfly *Idea leuconoe* (Erichson; Lepidoptera: Nymphalidae), which relies on PA such as parsonsianine to locate oviposition sites (Honda et al. 1997). However, at least for some other lepidopteran PA-specialists, oviposition could be mediated by synergistic effects between PA and other host-plant compounds. For instance, in experiments with the cinnabar moth, *Tyria jacobaeae* (Linnaeus; Lepidoptera: Erebidae) oviposition instances on substrates treated with PA-containing plant extracts were significantly higher than instances on substrates with pure PA. In addition, minute molecular differences between different PA such as the absence of a hydroxy group proved to be crucial for oviposition choice, suggesting a fine-tuned niche differentiation for this lepidopteran specialist (Macel and Vrieling 2003).

## Encoding taste valence and its role in associative learning

**Fig. 2:**
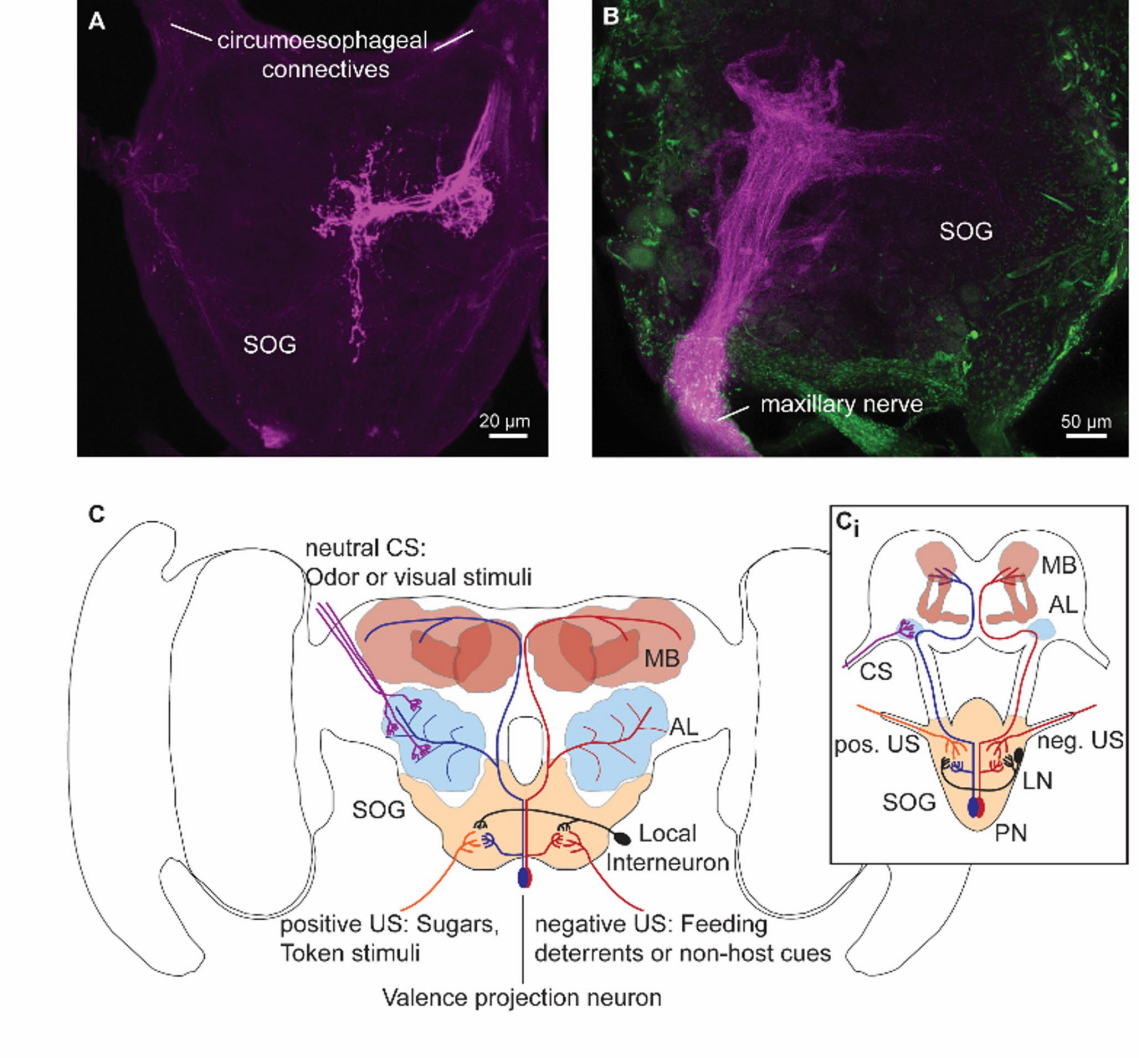
Gustatory input into the suboesophageal ganglion and its proposed processing pathway. **A** Backfill of the neurons innervating one pair of styloconic sensilla in a *Pieris brassicae* caterpillar (scale bar: 20 μm). **B** Backfill of one proboscis half in an adult *Manduca sexta* male moth (scale bar: 50 μm). Tetramethylrhodamine-labeled dextran was used for the anterograde staining in both cases (magenta). To-Pro3 Iodide (green) was used as background staining in the adult moths. **C** Hypothetical neuronal pathway linking tastants with a positive or negative valence (unconditioned stimulus) to a neutral odor or visual stimulus (conditioned stimulus) during associative learning in adult lepidopterans. Note that the visual pathway is not shown. **C**_**i**_ Hypothetical neuronal pathway linking tastants with different valences to neutral stimulus in caterpillars. *AL* Antennal lobe (blue), *CS* Conditioned stimulus, *MB* Mushroom body (red), *US* Unconditioned stimulus, *SOG* Suboesophageal ganglion (light brown), *LN* Local interneuron (black), *PN* Projection neuron (red and blue)

Tastants often carry an innate valence for an animal. This valence can be positive such as sugars or amino acids during feeding or token stimuli during host-plant choice, but tastants can also have a negative valence, acting as feeding or oviposition deterrents. Due to this innate value for the animal, tastants often act as positive or negative stimuli, i.e. the unconditioned stimulus, during associative learning (Haverkamp and Smid 2020; Hoedjes and Smid 2014). Learning and memory formation has been studied in moths and butterflies in some detail, using not only appetitive food rewards (Blackiston et al. 2011; Kelber 2003; Riffell et al. 2013), but also aversive taste stimuli (Dacks et al. 2012; Manel et al. 2025; Smallegange et al. 2006) and oviposition cues (Nataraj et al. 2021; Papaj 1986; Peftuloglu et al. 2024). Nevertheless, we know rather little about the neuronal pathway in the SOG that processes the taste information coming from the sensory organs (Fig. 2A, B), and that might contribute to establishing the valence of a certain taste compound.

In adult *Heliothis virescens* moths Central Gustatory Neurons (CGN) in the SOG generally show broad arborization patterns (Kvello et al. 2010). These CGNs often respond to the input of several different appendages such as the proboscis, antennae or tarsi, indicating that there is little conservation of the spatial information by these neurons. However, these neurons responded with different temporal firing patterns to the input from different appendages, for example burst-firing in response to proboscis sucrose neurons and phasic responses to tarsal sucrose neurons. These different response patterns could indicate that CGNs are involved in the processing of multiple appendage-specific behaviors (Kvello et al. 2010). Besides being responsive to information from multiple appendages, CGNs in both *H. armigera* caterpillars and *H. virescens* moths as well as adult *M. sexta* moths often respond broadly to several different tastants from several chemical and behavioral categories, suggesting that the valence of a taste stimulus is encoded in a complex across-fiber pattern rather than distinct labeled lines (Kvello et al. 2010; Reiter et al. 2015; Sun et al. 2022).

It is currently unknown how the valence computed in the SOG is linked to an another stimulus (i.e. the conditioned stimulus) during learning in Lepidoptera (Fig. 2C). In honeybees and flies distinct taste projection neurons transmit the information about positive feeding stimuli or feeding deterrents to other brain areas such as the antennal lobe or the mushroom bodies (Hammer 1993; Kim et al. 2017). However, no second order neuron encoding the unconditioned stimulus during associative learning has so far been identified in Lepidoptera with certainty. The caterpillar of *H. armigera* has one projection neuron which receives input from the SOG and projects towards the mushroom bodies (Sun et al. 2022). This neuron responded to a feeding deterrent but not to sucrose and might therefore mediate a negative valence during associative learning in this caterpillar. In the honeybee Ventral Unpaired Medial Neurons (VUMs) which have their cell bodies close to the maxillary (VUMmx1) and mandibular nerve (VUMmd3) respond to sucrose as well as odors and project from the SOG to the mushroom bodies (Hammer 1993; Schröter et al. 2007). One of these neurons, VUMmx1, has been shown to link the reward signal to a novel odor during associative learning, using octopamine as its neurotransmitter (Hammer and Menzel 1998). A similar octopaminergic neuron projecting from the SOG to the mushroom body lobes has also been identified in the hawkmoth *M. sexta*, but nothing is known about the response pattern of this neuron (Dacks et al. 2005). Furthermore, dye injection into the SOG revealed that projection neurons from the SOG innervate specific groups of Kenyon cells in adult *M. sexta* (Farris et al. 2011). Combined, these studies suggest that also in Lepidoptera projection neurons directly transmit information about the positive or negative valence of taste stimuli from the SOG to the mushroom bodies (Fig. 2C), although their explicit role during associative learning remains to be explored.

On a behavioral level different tastants are known to induce associative memories to different degrees. The hummingbird hawkmoth *Macroglossum stellatarum* (Linnaeus; Lepidoptera: Sphingidae) for example develops a preference for yellow feeders when these are paired with sucrose over their innately preferred blue feeders if these are linked to glucose, indicating that sucrose carries a more positive valence than glucose for these moths (Kelber 2003). Interestingly the performance during differential training in *M. stellatarum* also depends on the negative tastant that is used during the training in combination with the sucrose reward. If quinine is used as negative reinforcement memory recall is lower than when citric acid or no negative reinforcement is used (Manel et al. 2025). These results might suggest different detection mechanisms for the two negative stimuli, but might also hint at a tastant-specific interaction between positive and negative reinforcement stimuli during learning and memory formation.

Similarly to the influence of different tastants on food-related learning, oviposition learning is also influenced by the concentration-specific valence of the token stimulus. *Pieris* butterflies, for example, learn a novel odor better if they are trained with the sinigrin concentration which is best detected by the taste neurons on their tarsi (Peftuloglu et al. 2024). Although this study makes a clear link between the detection of a token stimulus and memory formation, it remains to be investigated how the token stimulus is processed by the central nervous system of the butterfly.

## Conclusions and future perspectives

The sense of taste is of paramount importance for larval and adult lepidopterans alike, allowing them to locate, select and reject food sources and oviposition sites. A substantial number of morphological and anatomical studies have been published with the development of electron microscopy, describing taste organs and the sensory neurons therein. Development of neurophysiological techniques during the 20th century allowed researchers to better understand how the mechanisms of taste detection at the periphery work. Combining electrophysiological responses to plant chemicals with behavioral responses has provided some understanding of the ecological relevance of generalist deterrent receptors that seem to have a wide occurrence and of the ecological function of token stimuli for specific lepidopteran families. In addition to this, recent advances in molecular techniques allowed for the identification of genes coding for GRs in taste organs (Gomes et al. 2024). Information on the molecular basis of taste detection will likely enable us to get a better grasp on taste coding and on the molecular evolution of gustatory recognition of host-plant taxa (Cicconardi et al. 2025).

Despite all these major achievements, the study of the sense of taste in Lepidoptera still leaves plenty of room for future exploration: first, the function of several taste organs in both larvae and adults is still poorly understood (e.g. epipharynx and maxillary palps in larvae, proboscis and ovipositor in adults), mostly due to technical challenges posed by the location and small size of these taste sensilla. In addition, the identification of the individual neuron in a taste sensillum that is responding to a ligand has rarely been achieved. The combination of detailed analysis of the multi-neural responses to mixtures of ligands and activity-related labeling techniques are required to resolve this. Plants saps exuding from wounds caused by caterpillar feeding and flower nectars are complex mixtures of chemical compounds and few studies have been done on the multi-neural activity they elicit (van Loon et al. 2008). In addition to neurophysiological recordings and activity-related labeling, nowadays molecular tools have expanded our range of study methods substantially (Wang et al. 2025). While the number of known genes coding for GRs in Lepidoptera is growing, little information is available on the receptor proteins themselves and on the molecular properties determining ligand binding. Lastly, through the combination of genomics and bioinformatics inferences about coevolutionary phenomena can be made, ultimately leading to a deeper understanding of the evolutionary pressures that shaped taste detection systems in Lepidoptera and their adaptation to specific ecological niches.

## Data Availability

No datasets were generated or analysed during the current study.
